# Real, Idealistic or Materialistic

**Published:** 1892-08

**Authors:** Hamilton Degraw


### WHICH, THE REAL, IDEALISTIC OR MATERIALISTIC?

BY HAMILTON DEGRAW, IN THE "MANIFESTO."

Depending largely upon the comparative development both intellectual and spiritual of the mind that is trying to solve the problem of what is life. To the understanding of a large majority of mankind, what their physical senses take cognizance of is the real, ignoring or not knowing that back of all physical manifestations or materializations there is a "great first cause." Omnipotent, omnipresent, unchangeable! While material forms are evanescent and constantly breaking up, disorganizing and reorganizing, not destroyed but assuming new forms, the idealistic or spiritual motive power that is making these changes in the material world is unchangeable.

To the architect that has evolved in his mind the form of an object to be materialized in wood, stone, iron or what not, which is the real? when perfected and fully evolved in his own interior consciousness, fire and flood may destroy and pulverize the outer manifestation yet the ideal remains intact, perfect in the mind's eye, ready again to be brought out in tangible form.

The farther removed from the inertia that characterizes the lowest forms of inorganic matter the more potent and powerful they become; so subtle that they cannot be viewed with our material vision and developing a power by which grosser forms of matter are moved irresistibly.

The electrical forces that man has harnessed and made to do him service are the most subtle and powerful of any elements that he has been able to grasp and retain. But who can say that it is the ultimate? may not forces be unfolded that in point of energy and power far transcend any as yet known? Like the sunlight through a prism the red rays are the crudest and ascending to the violet which is the most refined, and clairvoyant minds say they can see other colors that are too refined for our material vision to view.

"Beware when the great God lets loose a thinker on this planet." What does he do? thinks. Whoever saw a thought only as it has been materialized? All the acts of men in the outward form are but materialized thought. The mighty universe itself is but an evolved thought of the Infinite mind. The materialist insists upon the Infinity of matter, we upon the Infinity of mind which created matter, and as there cannot be two infinities, hence as a logical conclusion matter is finite, a created substance evolved from mind.

The world is prone to laugh at the so-called dreamers or visionary minds, who, living largely in the ideal are more in the future that the present. But as the wheels of time roll on, these become the practical workers, moving constantly ahead, planning and making the way easier for oncoming generations.

We have constantly before us an indisputable array of facts proving the reality of mind and the secondary position that matter the offspring of mind takes in the universe.

The series of undisputed victories of the mind of man over the forces of nature, in mastering the elements that have long evaded his grasp and compelling them to do him service, and the field is constantly widening in which to give scope to the latent but awakening powers that in time will give him complete control over every thing below him, is conclusive proof of the spirit's supremacy.

Still further proof lies in the fact that the soul is dissatisfied with its present environments and is placing its ideal always ahead of its reality, and in struggling to attain to its ideal is constantly placing it still further ahead and so on in infinite progression.

As parallel lines never meet, so the material never reaches the spiritual, but is always in a position of dependence to it, moving as a secondary, subject to the primary or first cause.

If such mighty results can be brought out of thought, that mankind are told to beware when a thinker is let loose to project his thoughts upon the race, who can measure the concentrated power developed when the whole race are thinkers and their united thought is used for the higher culture of the world. The alacrity with which progressive movements will take place cannot be comprehended at present.

Awakening from the slumber of ages mankind are virtually renewing their youth, casting off the swaddling clothes of spiritual infancy and assuming the proper dignity of developed men and women. Does this come from merely physical culture? By no means.

Though it is important to have a healthy body as the medium for the mind to act through, yet if the materialistic is the real, then the ox is equal to the man. Why do we feel the necessity of having an ideal in life, a something that is above the common condition of mortality? A holy of holies, a shrine at which the soul can worship and do homage to its ideal? Why do we embody this ideal in many instances in human form, some soul at whose feet we feel a pleasure in sitting and enjoying a holy communion away from and far above the material environments that are around us, and feel in the presence of such souls that holy "peace that passeth all understanding, and which the world can neither give nor take away." Why? because material conditions however perfect they may be fail to satisfy the spiritual perceptions, and when we see the embodiment of that ideal our souls instinctively know it, we need no herald to proclaim the truth. Why? because the love awakened in the soul by the contemplation of the perfect wishes to share unselfishly that which it possesses.

With this view of life there is awakened in the soul powers not dreamed of by the materialistic earth-bound, who like the man with the mud rake was constantly looking downward, his sense attracted to that which is beneath him instead of that which is above. The fact that a few controlling minds can sway the mass of mankind and move them as mere automatons, subject to their mental power is one more testimony given to substantiate the foregoing facts.

How soon the soul when deprived of the outward instinctively retires to the inner temple and draws from that hidden fountain vitalizing forces that in times of material prosperity were buried beneath the engrossing thoughts of worldly gain. Greater enjoyment is felt in the anticipation of some pleasure than in its material actualization. The struggle to gain a competence in worldly things is to the majority more invigorating and inspiring than its use after acquired. The former acting upon the mental forces, and the latter appealing to the bodily senses.

While it is important for the cultivation of the intellectual and spiritual nature that outward conditions should be harmoniously adjusted, the power of mind over materiality lies in the fact that it is able to compel many times an adjustment of outward conditions when to the materialistic view it seems impossible, as the rising from a bed of sickness when a great emergency demands vigorous action: the soul asserts its supremacy and compels from the body the homage due from the inferior to the superior.

When to the soul has been opened these heavenly portals revealing to its understanding the fact that its real life is not in the material, but spiritual realm, possibilities of growth are opened that to the materialistic mind are hid like precious treasures under large accumulations of earth.

In the realm of invention back of all visible signs is the realm of pure thought. Why does the inventor strive and agonize and labor, for what?

not to build the material form but to bring out the ideal, to perfect the mental conception, to evolve from chaos the idea. Then the rest is comparatively easy. Finite can never grasp the Infinite, and secondary can never assume the place of the primary cause.

Down in the interior ocean reigns a perpetual calm, so in the inner soul, the real, the pure idealistic is never disturbed by the exterior conflict that at times rages in the outer temple of life, because the nearer we approach the fountain of real life the more harmonious the adjustment. To live in the world of causes does not imply a neglect of the duties of life in the one of effects: for, "to be faithful with the unrighteous Mammon" prepares the soul to enjoy the true riches. Jesus prayed that his disciples might be saved from the world, not taken out. Lifted above the material where bondage to the physical senses holds the soul, into one where perfect freedom is attained. When this condition becomes the conscious inheritance of life it works a purifying process in all the faculties eliminating the crudities from our being that prevent us from enjoying the "communion of saints," intensifying our loves and making us more like the real, the ideal.

